# Xenobiotic Sulforaphane in Head and Neck Cancer: Beyond the Nrf2 Pathway

**DOI:** 10.3390/jox16030099

**Published:** 2026-06-01

**Authors:** Alessandro Polizzi, Rossella Rotondo, Sabrina Donati Zeppa, Monia Cecati, Magdalena Smolik, Valentina Schiavoni, Angelo Montana, Valentina Pozzi, Davide Sartini, Roberto Campagna, Gaetano Isola

**Affiliations:** 1 Department of General Surgery and Surgical-Medical Specialties, School of Dentistry, University of Catania, 95124 Catania, Italy; alessandro.polizzi@phd.unict.it (A.P.); gaetano.isola@unict.it (G.I.); 2 Department for the Promotion of Human Science and Quality of Life, San Raffaele Roma University, 00166 Rome, Italy; rossella.rotondo@uniroma5.it (R.R.); sabrina.donatizeppa@uniroma5.it (S.D.Z.); monia.cecati@uniroma5.it (M.C.); 3 IRCCS San Raffaele Roma, 03043 Cassino, Italy; 4 Department of Toxicology, Jagiellonian University Medical College, Medyczna 9, 30-688 Kraków, Poland; magdalena.smolik@uj.edu.pl; 5 Department of Clinical Sciences, Polytechnic University of Marche, 60126 Ancona, Italy; v.pozzi@univpm.it (V.P.); d.sartini@univpm.it (D.S.); 6 Department of Biomedical Sciences and Public Health, Polytechnic University of Marche, 60126 Ancona, Italy; a.montana@univpm.it

**Keywords:** sulforaphane, head and neck cancer, Nrf2

## Abstract

Head and neck cancers (HNCs) represent a major global health burden and remain associated with substantial morbidity and limited therapeutic options, particularly in advanced or recurrent disease. Increasing interest has focused on naturally derived bioactive compounds with potential chemopreventive and therapeutic properties. Sulforaphane, a dietary xenobiotic isothiocyanate derived from glucoraphanin in cruciferous vegetables, has attracted attention due to its ability to modulate redox balance, epigenetic regulation, and multiple oncogenic signaling pathways. This manuscript reviews current evidence regarding the biological effects of sulforaphane in HNCs. Particular attention is given to the molecular mechanisms underlying its modulation of the nuclear factor erythroid 2-related factor 2 (Nrf2) pathway, a key regulator of cellular antioxidant and detoxification responses that can be activated by sulforaphane. Several studies indicate that sulforaphane can inhibit tumor growth through several mechanisms beyond Nrf2 activation, including induction of apoptosis, cell cycle arrest, epigenetic modulation, and suppression of oncogenic signaling pathways. In addition, sulforaphane has been shown to enhance the efficacy of conventional treatments, including chemotherapy, radiotherapy, and photodynamic therapy. Overall, the literature suggests that sulforaphane may represent a promising chemopreventive or therapeutic adjunct in HNC, although further clinical investigation is required to clarify its translational potential.

^‡^ These authors share last authorship.

## 1. Head and Neck Cancer

Head and neck cancers (HNCs) collectively represent a major global oncological burden, ranking among the most frequently diagnosed malignancies worldwide. Recent epidemiological estimates indicate that these tumors account for about 890,000 new diagnoses and over 450,000 deaths every year, with projections suggesting a substantial increase in incidence in the coming decades [1]. By 2030, the global number of new cases is expected to exceed one million per year, reflecting both demographic changes and evolving exposure to risk factors. The geographical distribution of head and neck squamous cell carcinoma (HNSCC) varies considerably with particularly high incidence rates observed in regions such as Southeast Asia and parts of Oceania, where exposure to region-specific carcinogens, including areca nut and betel quid, remains frequent [2]. In contrast, the rising incidence in North America and Western Europe has been largely attributed to increasing rates of oropharyngeal infection with oncogenic human papillomavirus (HPV) [3].

Head and neck squamous cell carcinoma (HNSCC) originates in the epithelial tissues lining the upper aerodigestive tract, which includes the oral cavity, pharynx, and larynx, and accounts for approximately 90% of all HNCs [4]. In particular, oral squamous cell carcinoma (OSCC) represents 90% of all oral malignancies, and about 30% of all HNSCCs. The disease exhibits marked demographic variability: men are disproportionately affected, with a two- to four-fold higher risk compared to women, largely reflecting differences in exposure to modifiable risk factors [5]. Furthermore, the age at diagnosis differs according to etiological subtype. Tumors associated with traditional carcinogens such as tobacco and alcohol are typically diagnosed in older subjects, whereas virally driven cancers, particularly HPV-positive oropharyngeal carcinomas and Epstein–Barr virus (EBV)-associated nasopharyngeal carcinomas, tend to present at a younger age.

The etiology of HNSCC is multifactorial and encompasses environmental, lifestyle, infectious, and genetic determinants. Tobacco smoking and excessive alcohol consumption remain the most significant and widely distributed risk factors, with combined exposure conferring a dramatically elevated risk of disease development. Additional contributors include poor nutritional status, inadequate oral hygiene, and exposure to environmental pollutants, particularly in rapidly industrializing regions. In certain populations, the habitual use of areca nut-containing products is strongly linked to oral cavity carcinogenesis. Viral infections also play a critical role since persistent HPV infection is a well-established driver of oropharyngeal cancers, while EBV is implicated in nasopharyngeal carcinoma. Notably, HPV-positive HNSCC represents a distinct clinical entity characterized by improved responsiveness to therapy and more favorable prognosis compared to HPV-negative disease [6].

Genetic susceptibility further modulates individual risk [7]. Rare inherited disorders such as Fanconi anemia, characterized by defects in DNA repair pathways, confer a dramatically increased likelihood of developing HNSCC [8,9]. Additionally, polymorphisms in genes related to carcinogen processing and immune system regulation may affect the individual susceptibility to cancer, highlighting the complex interplay between host genetics and environmental exposures [10,11].

Despite advances in diagnostic and therapeutic approaches, the overall prognosis for HNSCC patients has improved only modestly over the past several decades. Population-based data indicate a gradual increase in 5-year survival rates; however, this improvement is largely attributable to the rising proportion of HPV-associated cases rather than significant breakthroughs in treatment for HPV-negative disease [12,13]. Standard therapeutic strategies, which include surgery, radiotherapy, and chemotherapy, are often employed in combination, particularly for advanced-stage tumors [14,15,16]. While these approaches can achieve locoregional control, they are frequently associated with substantial treatment-related morbidity, including long-term impairments in speech, swallowing, and overall quality of life [17,18]. Nevertheless, patients with recurrent or metastatic disease continue to have limited therapeutic options and poor survival outcomes [19]. These observations underscore the urgent need for more effective and less toxic therapeutic strategies.

In this setting, growing attention has focused on developing targeted, mechanism-driven strategies that address both intrinsic tumor alterations and the surrounding tumor microenvironment [20,21,22]. Molecular therapies aimed at dysregulated signaling pathways, epigenetic modifications, and tumor–stroma interactions hold promise for improving clinical outcomes [23,24,25,26,27,28]. Moreover, naturally derived bioactive compounds, including sulforaphane, have gained attention as promising agents for cancer prevention and treatment, owing to their capacity to modulate redox balance, epigenetic regulation, and key oncogenic pathways involved in HNSCC pathogenesis [29,30].

Based on this background, the present review adopts a hypothesis-driven approach. We hypothesize that sulforaphane activity in HNC is not limited to canonical Nrf2 activation, but reflects a context-dependent network of Nrf2-dependent and Nrf2-independent mechanisms that may influence chemoprevention, established tumor biology, treatment sensitization, antitumor immunity, and treatment-related toxicity. This framework guides the evaluation of the available evidence and its translational limitations.

## 2. Materials and Methods

This manuscript was designed as a narrative review aimed at summarizing the biological, pharmacological, and translational evidence on sulforaphane in head and neck cancer. A literature search was performed using PubMed/MEDLINE. The search strategy combined keywords and Boolean operators as follows: (“sulforaphane”) AND (“head and neck cancer” OR “HNC” OR “head and neck squamous cell carcinoma” OR “HNSCC” OR “oral squamous cell carcinoma” OR “OSCC” OR “oral cancer”). All original articles were selected if they addressed sulforaphane in relation to HNC biology, cancer-related molecular mechanisms, treatment response, pharmacokinetics, pharmacodynamics, bioavailability, safety, or translational development.

## 3. Sulforaphane: Chemical and Medicinal Chemistry Aspects

Plants belonging to the Brassicaceae family represent a rich dietary source of bioactive phytochemicals with well-documented health-promoting properties. These include polyphenols, vitamins such as ascorbic acid, essential micronutrients, and, notably, glucosinolates and their bioactive derivatives. Commonly consumed cruciferous vegetables, such as *Brassica oleracea* varieties including broccoli, cabbage, cauliflower, and kale, are particularly abundant in glucoraphanin, a major aliphatic glucosinolate predominantly localized in the aerial tissues of the plant, including florets and seeds [31].

Glucoraphanin itself is chemically stable and biologically inactive; however, upon mechanical disruption of plant tissue (e.g., chewing or food processing), it is exposed to the endogenous enzyme myrosinase, a β-thioglucosidase that catalyzes its hydrolysis. This enzymatic reaction results in the release of glucose and the formation of the unstable intermediate aglucone, which undergoes spontaneous rearrangement to yield several biologically active compounds, among which sulforaphane (1-isothiocyanato-4-(methylsulfinyl)butane) is the most extensively studied [32]. Sulforaphane is a sulfur-containing isothiocyanate recognized for its potent ability to modulate cellular defense mechanisms, particularly through the induction of phase II detoxification enzymes and antioxidant responses [33].

In humans, sulforaphane can be delivered either directly in its active form or indirectly via glucoraphanin-containing preparations. Notably, humans lack endogenous myrosinase activity; therefore, conversion of glucoraphanin to sulforaphane relies on plant-derived enzyme activity or, alternatively, on hydrolytic activity provided by the gut microbiota. The efficiency of this conversion is highly variable and influenced by multiple factors, including dietary habits, antibiotic exposure, and overall microbiome composition [34]. Consequently, inter-individual differences in sulforaphane bioavailability are considerable when glucoraphanin is used as a precursor [31,35].

Following absorption, sulforaphane undergoes extensive metabolism via the mercapturic acid pathway, leading to the formation of dithiocarbamate conjugates that are ultimately excreted in urine [31]. Sulforaphane can undergo reversible redox transformations to form related compounds such as erucin, while its precursor glucoraphanin is biosynthetically related to glucoerucin. Pharmacokinetic studies indicate that sulforaphane is rapidly absorbed and cleared, with typical urinary excretion of at least 70% of the dose, with relatively consistent excretion profiles across individuals, whereas glucoraphanin conversion is slower and exhibits greater variability [36].

Beyond these well-established biosynthetic and pharmacokinetic aspects, several chemical features of sulforaphane deserve specific consideration because they may influence its biological activity, experimental reproducibility, and translational development.

**Figure 1:**
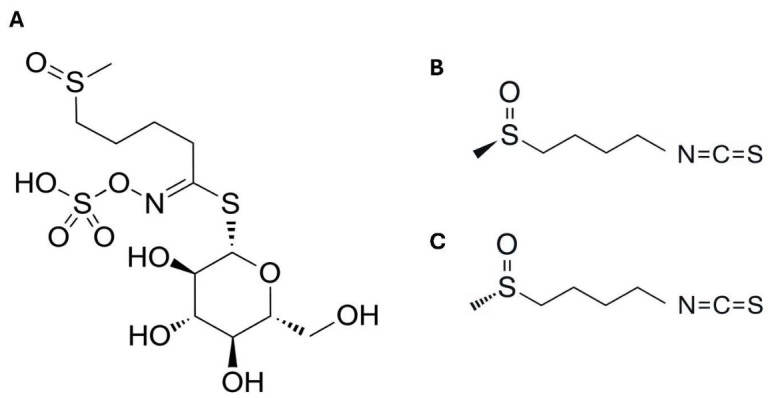
Chemical structure of glucoraphanin (**A**), (*R*)-sulforaphane (**B**) and (*S*)-sulforaphane (**C**).

From a stereochemical perspective, sulforaphane is a chiral sulfoxide whose stereogenic center is located at the sulfur atom, giving rise to the (*R*)- and (*S*)-enantiomers (Figure 1) [37,38].

Although dietary sulforaphane has often been considered predominantly represented by the naturally occurring (*R*)-enantiomer, enantioselective analytical studies have shown that natural sources are not necessarily enantiomerically pure. Okada et al. [39] detected both sulforaphane enantiomers in broccoli and broccoli sprouts, with *S/R* ratios of 1.5–2.6/97.4–98.5% in broccoli florets, 5.0–12.1/87.9–95.0% in broccoli stems, 8.3–19.7/80.3–91.7% in sprout leaves, and 37.0–41.8/58.2–63.0% in sprout stems. Therefore, the enantiomeric composition of sulforaphane may vary according to plant tissue and developmental stage, which is relevant when comparing dietary, botanical, and synthetic preparations [39]. This distinction is particularly relevant because many preclinical studies use synthetic racemic sulforaphane, whereas dietary exposure is generally enriched in the (*R*)-enantiomer. Enantioselective differences in biological activity have been reported. In particular, *R*-sulforaphane was shown to be a more potent inducer of carcinogen-detoxifying enzyme systems in rat liver and lung than the *S*-isomer, suggesting that the enantiomeric composition of sulforaphane preparations may affect pharmacodynamic interpretation [40].

Consistent with these considerations, synthetic preparations used in preclinical studies often consist of racemic mixtures, a point that should be considered when extrapolating experimental findings to dietary or botanical sulforaphane exposure [41].

The isothiocyanate group represents the key electrophilic pharmacophore of sulforaphane. This functional group enables sulforaphane to react with thiol-containing molecules, including glutathione and cysteine residues in regulatory proteins. Such reactivity provides a chemical basis for its metabolism through the mercapturic acid pathway and for the covalent modification of protein targets. These interactions are relevant not only for KEAP1-dependent Nrf2 activation, but also for Nrf2-independent effects involving redox modulation, apoptosis, inflammation, epigenetic regulation, and stress response signaling [42,43].

These chemical properties have stimulated medicinal chemistry approaches aimed at developing sulforaphane-like molecules with improved pharmacological profiles. Structural strategies include modification of the alkyl chain, oxidation state, and terminal substituents while retaining the isothiocyanate pharmacophore. Sulforaphane analogs containing sulfinyl, sulfanyl, sulfonyl, phosphonate, phosphinate, phosphine oxide, carbonyl, ester, carboxamide, ether, or additional isothiocyanate groups have been described, with the aim of tuning lipophilicity, electrophilicity, metabolic stability, and biological potency [42].

From a pharmacokinetic and pharmacodynamic perspective, the biological activity of sulforaphane is strongly influenced by its formulation, route of administration, and bioavailability. Free sulforaphane is rapidly absorbed after oral intake, whereas glucoraphanin requires conversion by active plant myrosinase or, when myrosinase is inactive, by the intestinal microbiota. Consequently, sulforaphane-rich preparations or glucoraphanin preparations containing active myrosinase generally provide faster and more efficient systemic exposure than glucoraphanin-only preparations. Food processing is also relevant, since heat treatment can reduce myrosinase activity and decrease sulforaphane formation from dietary precursors. After absorption, sulforaphane is rapidly conjugated with glutathione and metabolized through the mercapturic acid pathway, generating cysteinylglycine, cysteine, and N-acetylcysteine conjugates that are mainly eliminated in urine. These urinary dithiocarbamate metabolites are commonly used as biomarkers of systemic sulforaphane exposure. Pharmacodynamically, sulforaphane acts as a small electrophilic molecule whose isothiocyanate group modifies thiol-containing targets, including glutathione and cysteine residues in regulatory proteins. This explains its ability to activate KEAP1/Nrf2 signaling, but also contributes to Nrf2-independent effects involving redox modulation, glutathione metabolism, histone deacetylase inhibition, inflammation, apoptosis, and stress response pathways. These pharmacokinetic and pharmacodynamic features are particularly relevant for HNC translation, because systemic exposure, local tissue availability, formulation, and delivery route may influence whether sulforaphane behaves primarily as a chemopreventive, cytoprotective, or therapeutic adjunct [44,45,46]. These pharmacokinetic and pharmacodynamic features provide the basis for interpreting the biological effects of sulforaphane in cancer models. Interest in sulforaphane as an anticancer molecule has increased substantially over recent decades, driven by accumulating evidence supporting its protective effects across a wide spectrum of chronic diseases, including cancer, metabolic disorders, and cardiovascular conditions [47,48]. Mechanistically, many of these effects are attributed to activation of the nuclear factor erythroid 2-related factor 2 (Nrf2) signaling pathway.

## 4. Nrf2

Nrf2, encoded by the *NFE2L2* gene, is a master transcriptional regulator of cellular redox homeostasis and adaptive stress responses [49]. Nrf2 is a member of the Cap’n’Collar (CNC) subgroup of basic leucine zipper (bZIP) transcription factors, a family that also comprises Nrf1, Nrf3, and NF-E2. Structurally, Nrf2 is characterized by the presence of seven highly conserved Nrf2-ECH homology (Neh) domains (Neh1–Neh7), each of which contributes to its finely tuned regulation at multiple levels, including protein stability, transcriptional activation, and interaction with regulatory partners [50]. The Neh1 domain contains the bZIP motif responsible for DNA binding and heterodimerization with small musculoaponeurotic fibrosarcoma (sMAF) proteins (MAFG, MAFK, and MAFF). This heterodimeric complex enables Nrf2 to recognize and bind antioxidant response elements (AREs) within the promoter regions of target genes [51]. The Neh2 domain plays a central role in controlling Nrf2 stability, as it contains the two key motifs ETGE and DLG that mediate high- and low-affinity interactions, respectively, with Kelch-like ECH-associated protein 1 (KEAP1), the primary negative regulator of Nrf2. Through these interactions, KEAP1 functions as an adaptor protein that recruits Nrf2 to a Cullin 3 (CUL3)-based E3 ubiquitin ligase complex, promoting its continuous ubiquitination and proteasomal degradation under basal conditions. Additional domains further refine Nrf2 activity. The Neh3, Neh4, and Neh5 domains facilitate transcriptional activation through interactions with coactivators, including CREB-binding protein (CBP), as well as other components of the transcriptional machinery. In contrast, the Neh6 domain contains redox-independent degron motifs (DSGIS and DSAPGS) that mediate KEAP1-independent degradation via β-transducin repeat-containing protein (β-TrCP), linking Nrf2 turnover to glycogen synthase kinase-3β (GSK-3β)-dependent signaling. The Neh7 domain negatively regulates Nrf2 activity through its interaction with retinoid X receptor alpha (RXRα), thereby providing an additional layer of transcriptional repression [50,52].

**Figure 2:**
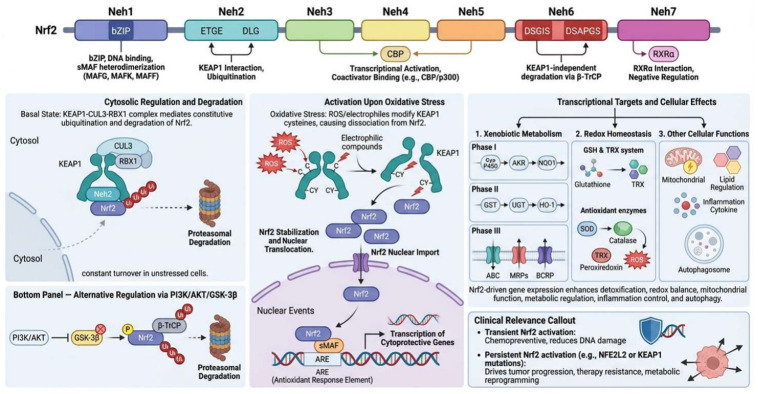
Structure, regulation, and cellular functions of the Nrf2 signaling pathway. The transcription factor Nrf2 contains seven functional domains (Neh1–Neh7) that coordinate its stability, protein interactions, and transcriptional activity. Under normal physiological conditions, KEAP1 promotes continuous ubiquitination and proteasomal degradation of Nrf2 in the cytoplasm. During oxidative stress, ROS modify critical cysteine residues in KEAP1, leading to the disruption the KEAP1–Nrf2 interaction and stabilizing Nrf2. Stabilized Nrf2 translocates into the nucleus, where it heterodimerizes with sMAF proteins and binds to AREs to induce transcription of cytoprotective genes.

Under physiological conditions, intracellular Nrf2 levels are maintained at low steady-state concentrations due to rapid proteasomal degradation mediated by the KEAP1–CUL3–RBX1 E3 ubiquitin ligase complex [53,54]. KEAP1 acts as a redox-sensitive sensor, containing reactive cysteine residues that respond to oxidative and electrophilic stress. Upon exposure to reactive oxygen species (ROS) or electrophilic molecules, critical cysteine residues within KEAP1 undergo covalent modification, leading to conformational changes that disrupt its ability to target Nrf2 for ubiquitination. As a result, newly produced Nrf2 avoids degradation, accumulates in the cytoplasm, and then translocates to the nucleus [55]. There, it heterodimerizes with sMAF proteins and binds ARE sequences, initiating the transcription of a broad array of cytoprotective genes (Figure 2) [56].

Beyond KEAP1-dependent regulation, Nrf2 activity is also modulated by signaling pathways such as phosphatidylinositol 3-kinase (PI3K)/Akt [57]. In this context, GSK-3β plays a pivotal role by phosphorylating Nrf2, thereby creating a recognition motif for β-TrCP and promoting its degradation through a CUL1-based E3 ligase complex. Activation of AKT results in inhibitory phosphorylation of GSK-3β, preventing Nrf2 phosphorylation and subsequent β-TrCP-mediated degradation. This crosstalk highlights how oncogenic signaling pathways can influence Nrf2 stability independently of KEAP1, a mechanism particularly relevant in cancer biology [50,58,59]. The transcriptional program governed by Nrf2 encompasses a wide spectrum of genes involved in cellular defense, detoxification, and metabolic adaptation. These include enzymes participating in phase I, II, and III xenobiotic metabolism. Phase I enzymes, such as members of the cytochrome P450 enzymes family, aldo-keto reductases (AKRs), carbonyl reductases, and aldehyde dehydrogenases, catalyze the initial modification of xenobiotics through oxidation, reduction, and hydrolysis reactions. In addition, Nrf2 induces cytoprotective enzymes such as NAD(P)H:quinone oxidoreductase 1 (NQO1), which mediates two-electron reduction of quinones and is functionally associated with Phase II detoxification pathways [60,61]. Phase II enzymes, including glutathione S-transferases (GSTs) and UDP-glucuronosyltransferases (UGTs), facilitate conjugation reactions that increase the solubility of metabolites, thereby promoting their elimination [62]. Phase III transporters, such as ATP-binding cassette (ABC) proteins, mediate the efflux of conjugated metabolites out of cells.

In addition to its role in xenobiotic metabolism, Nrf2 regulates genes involved in the synthesis and regeneration of key intracellular antioxidants, including glutathione (GSH) and thioredoxin (TRX) systems. It also controls the expression of enzymes responsible for the detoxification of reactive intermediates, such as superoxide dismutases (SODs), catalase, and peroxiredoxins. Through this coordinated transcriptional network, Nrf2 maintains redox balance and protects cells from oxidative damage [63].

## 5. Nrf2 Pathway in Cancer and Its Contribution to Sulforaphane Activity

Oxidative stress represents a key feature of cancer biology and plays a multifaceted role in tumor initiation and progression [64,65]. Cancer cells are characterized by elevated levels of ROS, including superoxide anion, hydroxyl radical, and hydrogen peroxide, which arise from mitochondrial dysfunction, oncogenic signaling, and metabolic reprogramming. At controlled levels, ROS function as signaling molecules that support proliferation, survival, and cellular adaptation; however, excessive accumulation can damage DNA, lipids, and proteins, thereby promoting genomic instability and mutagenesis [64,66]. To maintain redox homeostasis, cells rely on an intricate network of antioxidant defenses. These include enzymatic systems such as SODs, catalases, glutathione peroxidases, peroxiredoxins, thioredoxin systems, and various reductases, all of which cooperate to neutralize ROS and limit oxidative damage. In parallel, non-enzymatic antioxidants, including GSH, coenzyme Q, and lipoic acid, serve as critical buffers against oxidative stress. The equilibrium between ROS generation and antioxidant defenses is tightly controlled, and its impairment is strongly associated with the development of cancer [64,67].

Chronic inflammation further amplifies oxidative stress and contributes to tumor development by sustaining a microenvironment rich in cytokines, growth factors, and reactive species. Within this context, Nrf2 has emerged as a pivotal regulator of cellular defense mechanisms. Nrf2 activation enhances the transcription of genes involved in antioxidant responses and anti-inflammatory pathways, thereby protecting normal cells from oxidative damage and reducing the risk of malignant transformation. This protective role is supported by experimental evidence showing that genetic ablation of *NFE2L2* increases susceptibility to carcinogen-induced tumorigenesis [68,69].

Paradoxically, in established tumors, persistent activation of Nrf2 can confer a selective advantage to cancer cells [70]. By sustaining a reduced intracellular environment, Nrf2 enables malignant cells to tolerate elevated ROS levels associated with rapid proliferation and metabolic stress. Moreover, Nrf2 contributes to metabolic reprogramming by promoting anabolic pathways that support biomass accumulation and energy production, thereby facilitating tumor growth and invasiveness [71,72]. This functional switch underscores the context-dependent role of Nrf2 as both a tumor suppressor during early carcinogenesis and a tumor promoter in advanced disease.

At the functional level, persistent Nrf2 activity contributes to multiple hallmarks of cancer. It promotes cell proliferation by upregulating anabolic metabolism and modulating growth factor signaling pathways, including those driven by the epidermal growth factor receptor (EGFR) [73]. Nrf2 also promotes resistance to chemotherapy and radiotherapy by inducing the expression of detoxifying enzymes and drug efflux transporters, thereby reducing intracellular drug accumulation [74,75,76,77]. In addition, it upregulates antioxidant systems and anti-apoptotic proteins, such as members of the Bcl-2 family, enabling cancer cells to evade programmed cell death [78,79].

Beyond apoptosis resistance, Nrf2 contributes to additional adaptive mechanisms that favor tumor survival. It suppresses ferroptosis by maintaining glutathione homeostasis and regulating lipid peroxidation pathways, interferes with p53-mediated senescence programs, and protects telomeric DNA from oxidative damage [80,81,82,83]. Furthermore, Nrf2 has been implicated in promoting angiogenesis and facilitating epithelial–mesenchymal transition (EMT), partly through the downregulation of epithelial markers such as E-cadherin and the activation of invasion-associated transcriptional programs [84]. Its role in immune evasion is also increasingly recognized, as Nrf2 activation can inhibit antitumor immune responses by modulating cytokine production and reducing immune cell-mediated cytotoxicity [85,86,87].

**Figure 3:**
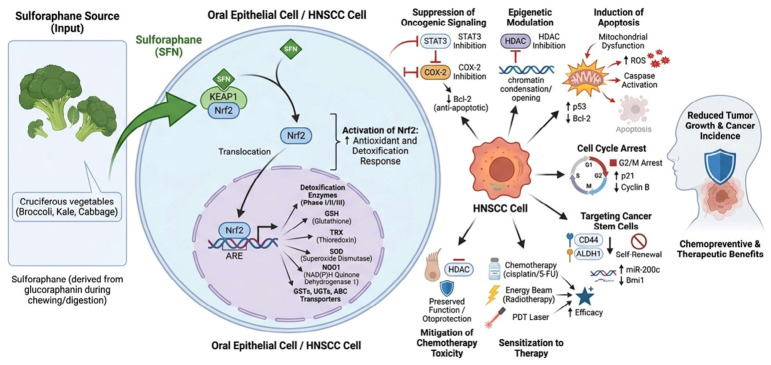
Proposed mechanisms underlying the preventive effects of sulforaphane in head and neck cancer (HNC).

Given its central involvement in cancer biology, the Nrf2 pathway has attracted considerable interest as a therapeutic target. However, its dual role presents a significant challenge; while activation of Nrf2 may be beneficial for cancer prevention and protection of normal tissues, inhibition of Nrf2 activity in tumor cells could enhance sensitivity to anticancer therapies [88,89]. A variety of pharmacological modulators, activators and inhibitors, have been investigated in this context. Nrf2 activators are often electrophilic compounds and exert their effects by modifying reactive cysteine residues in KEAP1, thereby disrupting its inhibitory function. Notable examples include sulforaphane, oltipraz, and dimethyl fumarate. These agents have demonstrated chemopreventive effects in preclinical models, largely through the induction of detoxification pathways (Figure 3) [90,91,92]. Future therapeutic strategies will likely require a context-dependent approach, aiming to selectively modulate Nrf2 activity according to disease stage and cellular context. Although sulforaphane is widely recognized as a potent activator of the Nrf2 pathway, the extent to which its anticancer effects in HNC are mediated by Nrf2 remains incompletely defined. Indeed, a substantial proportion of preclinical studies report antiproliferative and pro-apoptotic effects that appear to involve Nrf2-independent mechanisms, including modulation of ROS levels, mitochondrial dysfunction, epigenetic regulation, and inhibition of oncogenic signaling pathways such as STAT3 and COX-2. Moreover, in certain contexts, Nrf2 activation may exert cytoprotective effects that partially counterbalance sulforaphane-induced cytotoxicity. These observations highlight the complexity of sulforaphane’s mechanism of action and suggest that its biological effects in HNC cannot be solely attributed to Nrf2 activation.

These considerations have important implications for optimizing Nrf2 targeting in HNC. Nrf2 modulation should not be approached as a uniform strategy of pathway activation or inhibition, but rather as a context-dependent intervention guided by disease stage, tumor molecular background, treatment setting, and exposure duration. In normal or premalignant mucosa exposed to carcinogens, transient Nrf2 activation by sulforaphane may be advantageous by enhancing antioxidant and phase II detoxification responses, thereby limiting oxidative DNA damage and carcinogen-induced injury. Conversely, in established tumors, particularly in lesions characterized by constitutive Nrf2 activation, KEAP1/NFE2L2 alterations, high nuclear Nrf2 expression, or increased expression of Nrf2 target genes such as NQO1, GCLC, HO-1, AKRs, GSTs, and drug efflux transporters, further sustained Nrf2 activation could be undesirable because it may promote redox adaptation, metabolic reprogramming, apoptosis resistance, and resistance to chemotherapy or radiotherapy [93]. Therefore, future studies should define whether sulforaphane exposure produces transient adaptive Nrf2 activation or sustained cytoprotective signaling in HNC cells. From a therapeutic perspective, optimization may require patient selection according to Nrf2 pathway status, careful control of dose and schedule, and combination strategies that preserve the Nrf2-independent antitumor effects of sulforaphane, such as STAT3 inhibition, COX-2/Bcl-2 suppression, epigenetic modulation, ROS-mediated apoptosis, stemness inhibition, and immune modulation, while avoiding prolonged antioxidant protection of tumor cells. In Nrf2-addicted tumors, pharmacological inhibition of Nrf2 or targeting of downstream Nrf2-dependent vulnerabilities, including glutathione metabolism, thioredoxin-dependent redox control, drug efflux, ferroptosis resistance, and metabolic dependencies, may be more appropriate than additional Nrf2 activation. Thus, the optimal use of Nrf2-directed strategies in HNC should be biomarker-driven, stage-specific, and based on the distinction between chemopreventive activation in at-risk mucosa and therapeutic inhibition or limitation of sustained Nrf2 signaling in established tumors.

## 6. Sulforaphane in HNC

The anticancer effects of sulforaphane appear to involve multiple and partly overlapping mechanisms, including chemopreventive activity, modulation of oncogenic signaling, induction of cell cycle arrest and apoptosis, targeting of cancer stem cell traits, and enhancement of sensitivity to conventional therapies (Figure 3). The strength of the available evidence should be interpreted according to the experimental level at which sulforaphane has been investigated. Most data in HNC derive from in vitro studies using established cell lines, which are useful for defining molecular mechanisms but have limited translational weight because they do not reproduce tumor architecture, pharmacokinetics, immune interactions, or clinically achievable exposure. A smaller number of studies include in vivo models, such as carcinogen-induced oral tumorigenesis or xenograft models, providing stronger evidence for biological activity in a tissue context, although these models still do not fully recapitulate human HNC heterogeneity. Clinical evidence remains very limited and is currently restricted to pilot studies evaluating sulforaphane or broccoli sprout preparations in healthy volunteers, mainly assessing bioavailability and mucosal biomarker modulation rather than antitumor efficacy.

### 6.1. Preclinical Chemopreventive and Risk-Reduction Evidence

Bauman et al. [94] investigated the chemopreventive potential of sulforaphane in HNSCC using a combination of in vitro, in vivo, and translational human approaches. In vitro experiments were performed on a normal mucosal epithelial cell line (Het-1A) and multiple HNSCC cell lines, treated with sulforaphane at concentrations ranging approximately from submicromolar levels up to 40 µM, without combination with standard chemotherapeutic agents. Sulforaphane induced dose- and time-dependent activation of Nrf2 signaling, leading to upregulation of detoxification enzymes such as NQO1 and GCLC, which are involved in cellular defense against carcinogens. In parallel, sulforaphane promoted rapid dephosphorylation and inactivation of STAT3 independently of Nrf2, suggesting a dual mechanism involving both enhancement of cytoprotective pathways and suppression of oncogenic signaling. At higher concentrations, sulforaphane also induced apoptosis, with IC50 values around 21 µM, and this cytotoxic effect was enhanced upon Nrf2 silencing, indicating a protective role of Nrf2 against sulforaphane-induced cell death. In vivo, the study employed a carcinogen induced oral cancer model in C57BL/6 mice exposed to 4 nitroquinoline-1-oxide, with sulforaphane administered by oral gavage at 6 µmol three times per week. Sulforaphane treatment significantly reduced both the incidence and size of tongue tumors compared with controls, demonstrating a clear chemopreventive effect against carcinogen-driven oral tumorigenesis. Additionally, a pilot clinical study in healthy volunteers showed that oral administration or topical exposure to broccoli sprout extracts rich in sulforaphane or glucoraphanin resulted in measurable systemic bioavailability, while induction of the Nrf2 target gene NQO1 in oral mucosa was observed only in a subset of participants, indicating considerable interindividual variability in the mucosal response. Overall, this study demonstrates that sulforaphane exerts chemopreventive activity in head and neck cancer primarily through activation of Nrf2 dependent detoxification pathways and inhibition of STAT3 signaling, reducing tumor development in vivo and supporting its potential use in prevention strategies [94].

Notably, direct evidence supporting the involvement of Nrf2 in mediating the anticancer effects of sulforaphane in HNSCC remains limited, with most available studies focusing on Nrf2-independent mechanisms. Beyond its chemopreventive effects and its interaction with Nrf2-related signaling, several studies indicate that sulforaphane directly suppresses HNSCC cell growth by interfering with cell cycle progression and by activating apoptotic programs.

### 6.2. Therapeutic and Adjuvant Treatment Evidence in Established HNC

Krishnan et al. [95] investigated the antiproliferative and epigenetic effects of sulforaphane in oral squamous cell carcinoma using an in vitro model. The study was conducted on the human OSCC cell line UPCI-SCC-172, treated with sulforaphane at concentrations of 10, 20, and 30 µM for 24 and 48 h, without combination with conventional chemotherapeutic agents. Sulforaphane induced a significant dose- and time-dependent reduction in cell viability, with IC50 values of approximately 24 µM at 24 h and 14.3 µM at 48 h. A central finding of the study was the marked inhibition of histone deacetylase activity, reaching up to about 65% at 48 h, indicating a strong epigenetic modulatory effect. Functionally, sulforaphane exerted differential effects on cell cycle progression depending on concentration. At 10 µM, it induced a clear G2/M phase arrest accompanied by a reduction in the G1 population, whereas higher concentrations of 20 and 30 µM led to a prominent increase in sub G1 cells, indicating progression toward apoptosis rather than cell cycle blockade. This apoptotic effect was associated with increased intracellular ROS, loss of mitochondrial membrane potential, and activation of both intrinsic and extrinsic apoptotic pathways, as demonstrated by increased activity of caspase-3 and caspase-8. Overall, this study demonstrates that sulforaphane inhibits OSCC cell growth through HDAC dependent epigenetic regulation, leading to cell cycle arrest at lower doses and induction of apoptosis at higher doses, highlighting its potential as an epigenetic therapeutic agent in oral cancer [95].

Adtani et al. [96] evaluated the anticancer activity of sulforaphane in an in vitro model of oral squamous cell carcinoma using the human OECM-1 cell line. Cells were treated with a range of sulforaphane concentrations from 3.125 to 100 µM for 24 h, without combination with standard chemotherapeutic agents. Sulforaphane exerted a marked dose-dependent cytotoxic effect, with an IC50 of approximately 5.7 µM, indicating high potency at relatively low micromolar concentrations. Multiple complementary assays, including AO/PI staining, TUNEL, annexin V, and DNA fragmentation analysis, consistently demonstrated a significant induction of apoptosis following treatment. Mechanistically, sulforaphane induced mitochondrial dysfunction, as evidenced by a reduction in mitochondrial membrane potential detected by JC-1 staining, and promoted activation of the intrinsic apoptotic pathway. Western blot analyses revealed dose-dependent upregulation of pro-apoptotic markers such as Bax, caspase-3, caspase-9, PARP, cytochrome c, and the tumor suppressor p53, alongside downregulation of anti-apoptotic proteins including Bcl-2 and Smad4. Cell cycle analysis showed an increase in the sub G0/G1 population, indicating apoptotic DNA fragmentation, with additional alterations in cell cycle distribution consistent with growth inhibition. Overall, this study demonstrates that sulforaphane potently induces apoptosis in OSCC cells through a p53-mediated, mitochondria-dependent pathway, highlighting its potential as a targeted pro-apoptotic agent in oral cancer [96].

Cho et al. [97] investigated the antitumor effects of sulforaphane in oral squamous cell carcinoma through both in vitro and in vivo approaches, focusing on apoptosis regulation via COX-2 signaling. In vitro experiments were conducted using the human OSCC cell lines KB and YD-10B, treated with sulforaphane at concentrations of 20 and 40 µM for 24 and 48 h, with IC50 values reported around 22 to 26 µM. No conventional chemotherapeutic agents were combined with sulforaphane. The compound induced a marked dose-dependent reduction in cell viability, as shown by trypan blue exclusion and MTT assays, and caused evident morphological changes consistent with cell death. Mechanistically, sulforaphane triggered apoptosis through a caspase-dependent pathway, demonstrated by increased annexin V positivity, accumulation of sub G1 population, and cleavage of PARP and caspase-3, with apoptosis being abrogated by the pan-caspase inhibitor z-VAD. Sulforaphane selectively downregulated COX-2 expression without affecting COX-1, and this was associated with decreased levels of the anti-apoptotic protein Bcl-2, suggesting a COX-2 dependent survival pathway. In vivo, the study employed a nude mouse xenograft model generated by subcutaneous injection of KB cells, and oral administration of sulforaphane at 50 µg/kg for 21 days significantly reduced tumor volume and weight, while increasing apoptotic cell death in tumor tissues as evidenced by TUNEL staining. Overall, this study demonstrates that sulforaphane exerts a potent antitumor effect in OSCC primarily by inducing caspase-dependent apoptosis through suppression of the COX-2 Bcl-2 axis, supporting its potential as a chemopreventive or therapeutic agent targeting inflammatory survival pathways in oral cancer [97].

Kim et al. [98] investigated the antiproliferative effects of sulforaphane in oral squamous cell carcinoma using both in vitro cell culture systems and an in vivo xenograft model. In vitro experiments were conducted on the human OSCC cell lines KB and YD-10B, treated with sulforaphane at concentrations of 20 and 40 µM for 12 h, without combination with conventional chemotherapeutic agents. Sulforaphane significantly reduced cell proliferation in a dose-dependent manner, as shown by both trypan blue exclusion and MTT assays, with approximately 58 percent and 46 percent growth inhibition at 40 µM in KB and YD-10B cells, respectively. Flow cytometric analysis demonstrated that sulforaphane induced a marked accumulation of cells in the G2/M phase, increasing this population from about 28% to 54% in KB cells and from 24% to 35% in YD-10B cells, indicating a strong cell cycle arrest effect. This arrest was associated with a significant upregulation of the cyclin dependent kinase inhibitor p21 and a concomitant downregulation of cyclin B, while cyclin A levels remained unchanged. Additionally, luciferase reporter assays confirmed that sulforaphane enhanced p21 promoter activity, supporting transcriptional regulation of this pathway. In vivo, a nude mouse xenograft model generated by subcutaneous implantation of KB cells was used, and oral administration of sulforaphane at 50 µg/kg three times per week resulted in increased p21 expression within tumor tissues, as evidenced by immunohistochemistry, consistent with prior evidence of tumor growth inhibition in this model. Overall, this study demonstrates that sulforaphane exerts its antitumor effects in oral cancer primarily through induction of G2/M cell cycle arrest mediated by upregulation of p21 and modulation of cell cycle regulatory proteins, supporting its potential as a molecular targeted agent in OSCC [98].

Chen et al. [99] investigated the antitumor activity of sulforaphane cysteine, a metabolite of sulforaphane, in oral squamous cell carcinoma using in vitro models. The study was performed on the human OSCC cell lines HSC-3 and SCC-9, treated with sulforaphane cysteine at concentrations ranging from 5 to 40 µM for 24 h, without combination with conventional chemotherapeutic agents. Sulforaphane cysteine induced a clear dose-dependent reduction in cell viability and promoted accumulation of cells in the sub-G1 phase, indicating cell cycle disruption associated with apoptotic processes. Consistently, annexin V assays demonstrated a significant increase in apoptotic cell populations, particularly at higher concentrations. Treatment led to downregulation of key inhibitors of apoptosis, including cIAP-1 and XIAP, and activation of the caspase cascade, as evidenced by increased cleavage of caspase-8, caspase-9, caspase-3, and PARP. Mechanistic analyses showed activation of MAPK signaling pathways, with a prominent role for JNK, as pharmacological inhibition of JNK attenuated caspase activation, whereas inhibition of ERK or p38 had no comparable effect. Overall, this study demonstrates that sulforaphane cysteine exerts potent cytotoxic effects in OSCC cells by inducing apoptosis through JNK-dependent activation of caspase pathways and modulation of anti-apoptotic proteins, supporting the relevance of sulforaphane metabolites as mechanistically active agents in oral cancer [99]. Taken together, these findings suggest that sulforaphane exerts robust antiproliferative effects in HNSCC models, mainly through G2/M cell cycle arrest and activation of apoptosis via mitochondrial dysfunction, ROS accumulation, caspase activation, and modulation of survival-related pathways such as COX-2/Bcl-2. These findings further support the notion that sulforaphane-induced cytotoxicity is largely mediated through mechanisms that are not directly linked to Nrf2 activation.

In addition to its effects on bulk tumor cell proliferation and survival, sulforaphane has also been reported to target cancer stem cell-associated phenotypes, which are considered relevant to tumor initiation, invasiveness, therapeutic resistance, and recurrence. Liu et al. [100] investigated the antitumor activity of sulforaphane against oral squamous cell carcinoma stem-like cells using both in vitro and in vivo models. In vitro, the study used sphere forming cancer stem-like populations derived from the human OSCC cell lines SAS and GNM, with normal oral epithelial SG cells included as a non-malignant control. Sulforaphane was tested at concentrations of 10, 20, 40, and 80 µM for viability assays, while most functional stemness and motility experiments were performed mainly at 20 and 40 µM. No conventional chemotherapeutic agents were combined with sulforaphane in this study. Sulforaphane reduced OSCC cancer stem cells (CSC) proliferation in a dose dependent manner, with more limited effects on normal oral epithelial cells, and significantly impaired secondary sphere formation, indicating suppression of self-renewal capacity. It also decreased two canonical stemness markers—namely, CD44 positivity and ALDH1 activity—and inhibited migration, invasion, and anchorage-independent colony formation. In vivo, tumor initiating activity was assessed in BALB/c nude mice xenografted subcutaneously with OSCC CSCs, and intraperitoneal administration of sulforaphane at 50 mg/kg reduced xenograft growth compared with vehicle-treated controls. Mechanistically, sulforaphane induced a dose-dependent upregulation of miR-200c together with downregulation of Bmi1, supporting a link between sulforaphane exposure and repression of the stemness program. Overall, this study shows that sulforaphane does not simply exert a generic cytotoxic effect in OSCC, but specifically targets the cancer stem-like compartment, limiting self-renewal, invasive behavior, and tumor initiation, thus supporting its potential relevance as a stemness-directed preventive or therapeutic strategy in oral cancer [100].

Elkashty et al. [101] investigated the effects of sulforaphane in combination with conventional chemotherapy in HNSCC using in vitro models. The study was conducted on the human HNSCC cell lines SCC12 and SCC38, treated with sulforaphane at concentrations ranging from 0.875 to 14 µM, with most functional assays performed at 3.5 µM, either alone or in combination with cisplatin at 0.1 to 2 µg/mL or 5-fluorouracil at 0.013 to 130 µg/mL. Sulforaphane alone reduced cell viability in a dose- and time-dependent manner, with IC50 values of approximately 3.8 µM in both cell lines. When combined with chemotherapy, sulforaphane significantly enhanced cytotoxicity, more than doubling the effect of cisplatin and increasing the efficacy of 5-fluorouracil up to tenfold, particularly at lower drug concentrations. The combined treatments also markedly reduced clonogenic capacity and impaired post-treatment DNA repair, indicating a sustained loss of proliferative potential. Apoptosis assays showed that sulforaphane increased apoptotic cell fractions and further potentiated chemotherapy-induced apoptosis, with associated upregulation of pro apoptotic genes such as Bax and caspase-3 and downregulation of the anti-apoptotic gene Bcl-2. Importantly, sulforaphane exhibited minimal cytotoxicity toward non-malignant cells, including primary fibroblasts and epithelial progenitors, at concentrations effective against cancer cells. Overall, this study demonstrates that sulforaphane acts as a potent chemosensitizer in HNSCC, enhancing the efficacy of cisplatin and 5 fluorouracil through increased apoptosis and inhibition of DNA repair, while maintaining a favorable selectivity profile [101].

Elkashty et al. [102] investigated the effects of sulforaphane on head and neck squamous cell carcinoma cancer stem cells using both in vitro and in vivo models. In vitro experiments were conducted on CD44^+^/CD271^+^ cancer stem cell populations isolated by FACS from the human HNSCC cell lines SCC12 and SCC38. Sulforaphane was tested across a range of concentrations up to 14 µM, with functional assays primarily performed at 3.5 µM, and used both alone and in combination with cisplatin at 0.25 to 2 µg/mL or 5-fluorouracil at 0.013 to 130 µg/mL. Sulforaphane alone reduced CSC viability in a dose- and time-dependent manner, with IC50 values of approximately 5 µM, and significantly impaired clonogenicity and sphere-forming capacity, indicating inhibition of self-renewal. When combined with chemotherapy, sulforaphane markedly enhanced cytotoxicity, approximately doubling the effect of cisplatin and increasing the efficacy of 5-fluorouracil up to tenfold, particularly at lower drug concentrations, and completely abolished colony formation. Apoptosis assays showed that sulforaphane increased early apoptotic cell fractions, with further enhancement in combination treatments, reaching about 70% with cisplatin and 60% with 5-fluorouracil. Sulforaphane modulated key pathways involved in stemness and survival, reducing the expression of SOX2, OCT4, and ALDH1A1, as well as components of the SHH-related axis including NOTCH1, SMO, GLI1, and BMI1, while promoting caspase-3 activation and decreasing Bcl-2 expression. In vivo, a xenograft model generated by orthotopic injection of SCC12 derived CSCs into nude mice showed that sulforaphane at 4 mg/kg, administered alone or with cisplatin at 3 mg/kg, significantly reduced tumor growth, with the combination producing the strongest inhibition without detectable systemic toxicity. Overall, this study demonstrates that sulforaphane effectively targets the cancer stem cell compartment and acts as a potent chemosensitizer, enhancing the efficacy of standard chemotherapeutic agents while sparing normal stem cells, supporting its potential use as an adjuvant strategy in HNSCC [102].

Beyond its effects on chemotherapy response and cancer stem cell-associated phenotypes, sulforaphane may also influence antitumor immunity and immune-checkpoint-based therapeutic responses. Although direct evidence in HNC remains lacking, recent data from NSCLC suggest that sulforaphane may interact with immune checkpoint pathways and CD8^+^ T-cell-mediated antitumor immunity. Li et al. [103] reported that sulforaphane synergized with PD-1 blockade in NSCLC models, with enhanced tumor suppression when combined with anti-PD-1 therapy and chemotherapy, and increased markers of CD8^+^ T-cell antitumor activity. In addition, sulforaphane has been shown to activate CD8^+^ T-cell antitumor responses by reducing myeloid-derived suppressor cells within the tumor microenvironment, and to enhance CAR-T cell antitumor activity through modulation of the PD-1/PD-L1 axis. Since PD-1/PD-L1 blockade is clinically relevant in HNC, these findings provide a rationale for investigating whether sulforaphane can modulate the immune tumor microenvironment or influence response to checkpoint inhibitors in HNC models. However, these data should currently be interpreted as hypothesis-generating, because sulforaphane–immune checkpoint interactions have not yet been directly validated in HNC [103,104,105].

Another recurring theme across preclinical studies is the ability of sulforaphane to enhance the response of HNSCC cells to conventional anticancer treatments, supporting a possible role as a sensitizing adjunct rather than only as a direct antitumor agent. Lee et al. [106] investigated the potential of sulforaphane to enhance the cytotoxic effects of photodynamic therapy in head and neck cancer using an in vitro model. The study was conducted on the human laryngeal squamous cell carcinoma cell line AMC-HN3, treated with sulforaphane at concentrations up to 31.3 µM, with 7.8 µM selected for combination experiments, and photodynamic therapy performed using the photosensitizer photofrin at concentrations ranging from 1.6 to 3.2 µg/mL followed by laser irradiation at 630 nm with an energy dose of 2.0 J/cm^2^. Sulforaphane alone induced a dose-dependent reduction in cell viability, while photodynamic therapy also produced cytotoxic effects in a concentration-dependent manner. Importantly, the combination of sulforaphane with photodynamic therapy significantly enhanced cytotoxicity compared to either treatment alone, leading to a more pronounced decrease in cell viability. This effect was associated with a marked increase in both apoptotic and necrotic cell populations, as well as a significant elevation in intracellular ROS levels. Mechanistically, the combination treatment strongly activated the caspase cascade, including caspase-3, caspase-8, caspase-9, and PARP cleavage, indicating involvement of both intrinsic and extrinsic apoptotic pathways. The use of sodium azide, a scavenger of reactive oxygen species, attenuated ROS generation, reduced apoptosis and necrosis, and inhibited caspase activation, confirming the central role of oxidative stress in mediating the observed effects. Overall, this study demonstrates that sulforaphane enhances the efficacy of photodynamic therapy in head and neck cancer cells by amplifying ROS-dependent apoptotic signaling, supporting its potential use as a sensitizing agent in combination treatment strategies [106].

Kotowski et al. [107] investigated the potential radiosensitizing effects of sulforaphane in HNSCC using in vitro models. The study was conducted on four human HNSCC cell lines—namely, SCC9, SCC25, CAL27, and FADU—treated with sulforaphane at concentrations ranging from 0 to 20 µM and subsequently exposed to ionizing radiation at doses of 2, 4, or 8 Gy. Sulforaphane alone induced a dose-dependent inhibition of cell proliferation, with IC50 values ranging approximately between 5 and 7 µM after 72 h. When combined with radiation, sulforaphane significantly enhanced growth inhibition compared to either treatment alone, with combination index analysis demonstrating a synergistic interaction across all cell lines. Clonogenic assays confirmed a marked reduction in long-term survival following combined treatment, indicating a sustained impairment of reproductive capacity. Apoptosis analysis showed that sulforaphane increased apoptotic rates at higher concentrations, although the addition of radiation did not further augment apoptosis compared to sulforaphane alone under the tested conditions. The expression of the anti-apoptotic proteins Akt and Mcl-1 was not significantly altered by treatment. Overall, this study demonstrates that sulforaphane enhances radiosensitivity in HNSCC cells primarily through inhibition of proliferation and clonogenic survival rather than through modulation of classical apoptotic pathways, supporting its potential role as a radiosensitizing agent in this malignancy [107].

**Figure 4:**
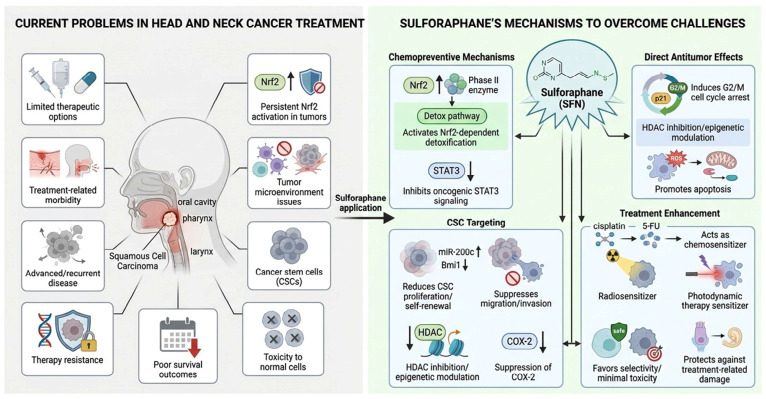
Overview of current challenges in HNC treatment and the mechanisms by which sulforaphane may overcome them.

Wang et al. [108] explored the biological effects of sulforaphane in both in vitro and in vivo models, primarily focusing on its interaction with cisplatin-induced toxicity rather than its direct antitumor activity. The in vivo component employed a rat model treated with cisplatin at 7 mg/kg twice daily for 7 days, with or without sulforaphane administered intraperitoneally at 30 mg/kg once daily. In vitro experiments were conducted using the murine auditory cell line HEI-OC1 and rat cochlear organotypic cultures exposed to cisplatin at 10 µM, in combination with sulforaphane at concentrations of 5, 10, and 15 µM. Although this study does not involve head and neck cancer models, it is relevant in the context of chemotherapy-related toxicity. Sulforaphane exerted a clear dose-dependent protective effect against cisplatin-induced cellular damage, as evidenced by improved cell viability, preservation of hair cell morphology, and increased Myosin VIIa-positive cells, as shown in the cochlear cultures. In HEI-OC1 cells, cisplatin reduced viability to approximately 42%, while co-treatment with sulforaphane significantly restored survival. Mechanistically, sulforaphane modulated epigenetic regulation by inhibiting the cisplatin-induced upregulation of HDAC2, HDAC4, and HDAC5, while restoring histone acetylation levels through recovery of H3-Ack9 expression. Notably, no significant effects were observed on HDAC7 or HDAC9. In vivo, sulforaphane attenuated cisplatin-induced functional damage, reducing ABR threshold shifts and outer hair cell loss. Overall, this study demonstrates that sulforaphane can mitigate cisplatin-induced cytotoxicity through modulation of histone acetylation pathways, suggesting a potential role as a protective adjunct during chemotherapy (Figure 4) [108]. Despite the promising preclinical evidence, the clinical translation of sulforaphane in HNC remains limited (Table 1). To date, clinical evidence is restricted to a small pilot study in healthy individuals, and no controlled clinical trials have evaluated its efficacy in patients with HNC. Several factors may contribute to this translational gap. Sulforaphane bioavailability shows substantial interindividual variability, particularly when administered as glucoraphanin-containing preparations that require enzymatic conversion by plant myrosinase or the gut microbiota. Moreover, sulforaphane is characterized by rapid metabolism and elimination, potentially limiting sustained tissue exposure. In addition, the concentrations required to achieve anticancer effects in preclinical models may be difficult to reproduce in vivo through dietary administration alone. Another important issue concerns the dual role of Nrf2 in cancer biology, since prolonged activation of the pathway may exert cytoprotective effects that could potentially favor survival of established tumor cells in certain contexts. Future translational strategies may therefore require optimization of formulation and delivery systems, improved standardization of sulforaphane preparations, identification of predictive biomarkers of response, and careful patient selection according to tumor molecular characteristics and Nrf2 pathway status. Overall, the available evidence indicates that the activity of sulforaphane in HNC extends beyond Nrf2 activation alone and involves a broader network of effects, including modulation of proliferation, apoptosis, stemness-related traits, and treatment responsiveness.

## 7. Safety, Toxicity, and Drug–Drug Interaction Considerations

Although sulforaphane is generally considered a dietary bioactive compound with a favorable safety profile, its potential therapeutic use requires careful evaluation of dose, formulation, exposure duration, and patient-specific risk factors. Most studies in HNC models have reported selective cytotoxicity toward malignant cells and limited toxicity toward non-malignant cells; however, these findings are largely preclinical and should not be directly extrapolated to clinical use. At pharmacological concentrations, sulforaphane may also affect non-tumor cell populations through mechanisms related to electrophilic stress, redox modulation, calcium signaling, and thiol reactivity.

One relevant safety concern is the ability of sulforaphane to induce eryptosis, a form of suicidal erythrocyte death characterized by cell shrinkage and phosphatidylserine exposure on the erythrocyte surface. Alzoubi et al. showed that exposure of human erythrocytes to sulforaphane at 50–100 μM for 48 h significantly decreased forward scatter, increased annexin V binding, elevated cytosolic Ca^2+^ activity, and enhanced ceramide formation, indicating stimulation of eryptosis [109]. Although these concentrations may exceed those typically achieved through dietary intake, this finding is relevant for therapeutic development because enhanced eryptosis could theoretically contribute to anemia, impaired microcirculation, or reduced erythrocyte lifespan in susceptible patients. Therefore, future translational studies should include hematological safety endpoints, particularly when high-dose or prolonged sulforaphane formulations are considered.

Drug–drug interactions represent another important issue. Sulforaphane and related isothiocyanates can modulate xenobiotic-metabolizing enzymes, including cytochrome P450 isoforms. Skupinska et al. [110] reported that sulforaphane and selected analogs inhibited benzo[a]pyrene-induced CYP1A1 and CYP1A2 activity in experimental cell models. Since CYP1A2 participates in the metabolism of several clinically used drugs, inhibition of this pathway could potentially alter the exposure or tolerability of CYP1A2 substrates, including drugs such as propranolol, cyclobenzaprine, and selected psychotropic agents [110]. In oncology patients, this issue may be particularly relevant because polypharmacy is common and supportive medications, analgesics, antidepressants, antiemetics, cardiovascular drugs, and anticancer agents may share CYP-mediated metabolic pathways.

These observations do not preclude the clinical development of sulforaphane, but they indicate that safety cannot be inferred solely from its dietary origin. Future studies should define the therapeutic window of sulforaphane, distinguish dietary exposure from pharmacological dosing, monitor hematological parameters, and systematically evaluate interactions with CYP-metabolized drugs. Until such evidence is available, sulforaphane should be considered an experimental adjunct rather than a clinically established therapeutic agent in HNC.

## 8. Future Directions

Future research on sulforaphane in HNC should move beyond descriptive preclinical evidence and address several translational priorities. First, additional studies are needed to define clinically relevant exposure levels. Most available data derive from in vitro models using micromolar concentrations of sulforaphane, whereas human evidence remains limited to bioavailability and biomarker studies in healthy volunteers rather than efficacy studies in HNC patients. Therefore, future investigations should clarify whether concentrations associated with antiproliferative, pro-apoptotic, stemness-targeting, or treatment-sensitizing effects can be achieved in oral and pharyngeal mucosal tissues through dietary, systemic, topical, or local delivery strategies.

Formulation and delivery require further optimization. Because sulforaphane bioavailability is influenced by glucoraphanin conversion, myrosinase activity, the gut microbiota, food processing, and rapid mercapturic acid metabolism, future studies should compare standardized free sulforaphane, glucoraphanin-plus-myrosinase preparations, stabilized formulations, and local mucosal delivery systems. In HNC, topical or locoregional delivery may be particularly relevant, as it could increase exposure at the oral or oropharyngeal mucosa while limiting systemic variability.

Future studies should adopt biomarker-driven designs. Given the context-dependent role of Nrf2, sulforaphane should not be investigated as a uniform Nrf2 activator across all disease settings. Instead, tumor Nrf2 pathway status, including KEAP1/NFE2L2 alterations, nuclear Nrf2 expression, and expression of Nrf2 target genes such as NQO1, GCLC, HO-1, AKRs, GSTs, and drug efflux transporters, should be assessed to distinguish tumors in which transient Nrf2 activation may be beneficial from those in which sustained Nrf2 signaling could promote cytoprotection and treatment resistance.

Additionally, the most promising therapeutic direction appears to be the use of sulforaphane as an adjunct to established treatments rather than as a stand-alone anticancer agent. Preclinical studies support potential combinations with cisplatin, 5-fluorouracil, radiotherapy, photodynamic therapy, and possibly immune checkpoint blockade. These combinations should be tested in more representative models, including three-dimensional cultures, patient-derived organoids, immune-competent animal models, and patient-derived xenografts. Particular attention should be given to whether sulforaphane enhances antitumor efficacy, reduces treatment-related toxicity, or both.

Finally, clinical translation will require carefully designed early-phase trials. Such studies should include pharmacokinetic and pharmacodynamic endpoints, standardized sulforaphane formulations, tissue and blood biomarkers, safety monitoring, hematological parameters, and systematic assessment of drug–drug interactions. At present, sulforaphane should be regarded as an experimental chemopreventive or therapeutic adjunct in HNC. Its future development will depend on demonstrating reproducible tissue exposure, identifying responsive molecular subgroups, confirming safety in patients receiving standard oncologic therapies, and establishing clinically meaningful benefit in controlled studies.

## 9. Conclusions

The available literature consistently indicates that sulforaphane exerts multiple antitumor activities in HNC through diverse molecular mechanisms. Across in vitro and in vivo models, sulforaphane has been shown to inhibit tumor growth by inducing apoptosis, promoting cell cycle arrest, modulating epigenetic regulators, and suppressing oncogenic signaling pathways such as STAT3 and COX-2. In addition, it targets cancer stem cell populations, reducing self-renewal capacity and tumor-initiating potential, and enhances the efficacy of conventional treatments including chemotherapy, radiotherapy, and photodynamic therapy. Sulforaphane has shown a favorable selectivity profile in several preclinical models, with limited toxicity toward selected non-malignant cells and potential protective effects against treatment-related damage; however, possible hematological toxicity, dose-dependent off-target effects, and drug–drug interactions require careful evaluation before clinical translation. Collectively, these findings support sulforaphane as a promising chemopreventive and therapeutic adjunct in HNC. However, current evidence remains predominantly preclinical, and controlled translational studies are required to determine whether sulforaphane can achieve reproducible tissue exposure, acceptable safety, and clinically meaningful benefit in HNC patients.

## Figures and Tables

**Table 1:** Summary of studies focused on the role of sulforaphane in HNSCC.

Type of Study	Model/Cell Lines	Sulforaphane (SFN) Concentration/Combination Strategy	Effects	Reference
In vitro	Het-1A; UMSCC-22A, UMSCC-1, Cal33, UPCI:SCC090 cell lines	SFN up to 40 µM (IC50 ~21 µM)	Nrf2 activation; increased detoxification enzymes; STAT3 inhibition; dose-dependent apoptosis	[94]
In vivo	C57BL/6 mice, 4NQO model	SFN 6 µmol (oral gavage, 3×/week)	Reduced tumor incidence and size (chemopreventive effect)	[94]
In vivo	Clinical trial (healthy volunteers)	Broccoli sprout extract	Systemic bioavailability; activation of Nrf2 target genes in oral mucosa	[94]
In vitro	UPCI-SCC-172 cell line	SFN 10–30 µM	HDAC inhibition; G2/M arrest (low dose); apoptosis via ROS and caspases (high dose)	[95]
In vitro	OECM-1 cell line	SFN 3.125–100 µM	Mitochondrial apoptosis (p53-dependent); ↑ Bax and caspases; ↓ Bcl-2	[96]
In vitro	KB, YD-10B cell lines	SFN 20–40 µM	Caspase-dependent apoptosis; ↓ COX-2 and Bcl-2	[97]
In vivo	KB xenograft, nude mice	SFN 50 µg/kg	Reduced tumor volume and weight; increased apoptosis	[97]
In vitro	KB, YD-10B cell lines	SFN 20–40 µM	G2/M cell cycle arrest; ↑ p21; ↓ cyclin B; proliferation inhibition	[98]
In vivo	KB xenograft, nude mice	SFN 50 µg/kg (3×/week)	Increased p21 expression; tumor growth inhibition	[98]
In vitro	HSC-3, SCC-9 cell lines	SFN 5–40 µM	Apoptosis via JNK and caspase activation; ↓ cIAP-1 and XIAP	[99]
In vitro	SAS cell line, GNM CSCs; SG normal epithelial cells	SFN 10–80 µM	Reduced cancer stem cell properties (self-renewal, CD44, ALDH1); decreased invasion; ↑ miR-200c	[100]
In vivo	CSC xenograft, nude mice	SFN 50 mg/kg	Reduced CSC-derived xenograft growth	[100]
In vitro	SCC12 and SCC38 cell lines	SFN 0.875–14 µM + cisplatin 0.1–2 µg/mL or 5-FU 0.013–130 µg/mL	Cytotoxicity; chemosensitization (enhanced cisplatin/5-FU efficacy); increased apoptosis	[101]
In vitro	SCC12 cell line, SCC38 CSCs; CD44+/CD271+	SFN up to 14 µM, mainly 3.5 µM + cisplatin 0.25–2 µg/mL or 5-FU 0.013–130 µg/mL	Reduced CSC viability and clonogenicity; chemosensitization; ↓ SOX2, OCT4	[102]
In vivo	SCC12 CSC orthotopic xenograft, nude mice	SFN 4 mg/kg alone or combined with cisplatin 3 mg/kg	Tumor growth inhibition; enhanced effect with cisplatin	[102]
In vitro	AMC-HN3 cell line	SFN 7.8 µM + photodynamic therapy with photofrin 1.6–3.2 µg/mL and 630 nm irradiation, 2.0 J/cm^2^	Enhanced photodynamic therapy; ↑ ROS; increased apoptosis and necrosis	[106]
In vitro	SCC9, SCC25, CAL27, FADU cell lines	SFN 0–20 µM + ionizing radiation 2, 4 or 8 Gy	Radiosensitization; reduced proliferation and clonogenic survival	[107]
In vitro	HEI-OC1 cell line; cochlear cultures	SFN 5–15 µM + cisplatin 10 µM	Protection against cisplatin toxicity; improved viability; HDAC modulation	[108]
In vivo	rat model	SFN 30 mg/kg + cisplatin 7 mg/kg	Protection against cisplatin-induced ototoxicity	[108]

## Data Availability

No new data were created or analyzed in this study.

## Abbreviations

The following abbreviations are used in this manuscript:

ABC	ATP-binding cassette
AKRs	aldo-keto reductases
AREs	antioxidant response elements
CBP	CREB-binding protein
CNC	Cap’n’Collar
CSC	cancer stem cell
CUL3	Cullin 3
EBV	Epstein–Barr virus
EGFR	epidermal growth factor receptor
EMT	epithelial–mesenchymal transition
GSH	glutathione
GSK-3β	glycogen synthase kinase-3β
GSTs	glutathione S-transferases
H_2_O_2_	hydrogen peroxide
HNCs	Head and neck cancers
HNSCC	head and neck squamous cell carcinoma
HPV	human papillomavirus
KEAP1	Kelch-like ECH-associated protein 1
Neh	Nrf2-ECH homology
NQO1	NAD(P)H:quinone oxidoreductase 1
Nrf2	nuclear factor erythroid 2-related factor 2
OSCC	oral squamous cell carcinoma
PI3K	phosphatidylinositol 3-kinase
ROS	reactive oxygen species
RXRα	retinoid X receptor alpha
sMAF	small musculoaponeurotic fibrosarcoma
SODs	superoxide dismutases
TRX	thioredoxin
UGTs	UDP-glucuronosyltransferases
β-TrCP	β-transducin repeat-containing protein
